# Supplementary material for: Identification and functional analysis of non-coding regulatory small RNA FenSr3 in Bacillus amyloliquefaciens LPB-18
Source: PeerJ. 2023 May 15;11:e15236. doi: 10.7717/peerj.15236 (PMC10194069; doi:10.7717/peerj.15236)
Supplement: Supplemental Information 4 [file peerj-11-15236-s004.zip › KO/CK-vs-T1_map/map05030.html]

KEGG PATHWAY: Cocaine addiction - Reference pathway


|  |  |
| --- | --- |
| **Cocaine addiction - Reference pathway** |  |

[
Pathway menu
| Organism menu
| Pathway entry
| Show description
| User data mapping
]

Drug addiction is a chronic, relapsing disorder in which compulsive drug-seeking and drug-taking behavior persists despite serious negative consequences.There is strong evidence that the dopaminergic system that projects from the ventral tegmental area (VTA) of the midbrain to the nucleus accumbens (NAc), and to other forebrain sites, is the major substrate of reward and reinforcement for both natural rewards and addictive drugs. Cocaine binds strongly to the dopamine-reuptake transporter, preventing the reuptake of dopamine into the nerve terminal. Because of this blocking effect, dopamine remains at high concentrations in the synapse and continues to affect adjacent neurons, producing the characteristic cocaine "high." Activated D1 receptor activates the PKA signaling pathway, and this pathway plays a critical role in mediating the behavioral responses to cocaine administration. Cocaine-induced neuroadaptations, including dopamine depletion, may underlie craving and hedonic dysregulation.

|  |  |  |
| --- | --- | --- |
| Reference pathway | 184% 150% 122% 100% 82% 67% 55% | 图片下载 |
